# CPLANE Complex and Ciliopathies

**DOI:** 10.3390/biom12060847

**Published:** 2022-06-17

**Authors:** Jesús Eduardo Martín-Salazar, Diana Valverde

**Affiliations:** 1 CINBIO, Biomedical Research Centre, University of Vigo, 36310 Vigo, Spain; edu19ms@gmail.com; 2 Galicia Sur Health Research Institute (IIS-GS), 36310 Vigo, Spain

**Keywords:** cilia, ciliopathies, CPLANE

## Abstract

Primary cilia are non-motile organelles associated with the cell cycle, which can be found in most vertebrate cell types. Cilia formation occurs through a process called ciliogenesis, which involves several mechanisms including planar cell polarity (PCP) and the Hedgehog (Hh) signaling pathway. Some gene complexes, such as BBSome or CPLANE (ciliogenesis and planar polarity effector), have been linked to ciliogenesis. CPLANE complex is composed of *INTU*, *FUZ* and *WDPCP*, which bind to *JBTS17* and *RSG1* for cilia formation. Defects in these genes have been linked to a malfunction of intraflagellar transport and defects in the planar cell polarity, as well as defective activation of the Hedgehog signalling pathway. These faults lead to defective cilium formation, resulting in ciliopathies, including orofacial–digital syndrome (OFDS) and Bardet–Biedl syndrome (BBS). Considering the close relationship, between the CPLANE complex and cilium formation, it can be expected that defects in the genes that encode subunits of the CPLANE complex may be related to other ciliopathies.

## 1. Introduction

The term “ciliopathies” emerged in 2006 [1] to describe a heterogeneous group of rare genetic disorders that share a common aetiology: defects in cilia and centrosome/basal body structure and/or function

Cilia are highly conserved organelles that project from the surface of almost all eukaryotic cells and have important roles primarily in sensory perception and cell motility [2]. Cilia are classified as motile and sensory or primary cilia [3]. Primary cilia are cell-cycle-associated organelles which have been described as small antennae protruding from the cell surface [4] and are present on almost all mammalian cell types, while motile cilia are assembled by certain cell types such as respiratory epithelia, sperm flagella and fallopian tubes [5].

Primary cilia detect and transduce extracellular signals, such as biochemical and mechanical stimuli, to regulate processes including differentiation and proliferation [6].

Since primary cilia were first discovered in chondrocytes in 1967 [7], an increasing number of studies have focused on exploring their functions, mainly their role in endocytosis, infiltration and apoptosis [8]. Initially, primary cilia had been considered unimportant vestigial structures. However, increasing evidence suggests that primary cilia are functional organelles that can not only detect chemical or mechanical signals in the extracellular environment [9], but can also regulate cell mitosis [10] and signal transduction [11].

The primary cilium is composed of nine microtubule doublets (9 + 0 structure), building the ciliary axoneme. This structure is covered by a membrane that is continuous with the plasma membrane, but it has a particular protein and lipid content that is essential for ciliary activity [12,13].

Primary cilia are dynamic organelles that are continually being assembled and reabsorbed in a process tightly coupled to the cell cycle. The assembly process, called ciliogenesis, is made up of several steps. The axoneme elongates from the basal body, which is the transformed mother centriole with distal and subdistal appendages. Cilia formation is initiated by the apical migration of the mother centriole to become the basal body, followed by extension of the axoneme microtubules, formation of the transition zone and growth of the cilium by the ciliary trafficking machinery such as intraflagellar transport [14,15]. There are different mechanisms and signalling pathways involved in cilia formation, two of the most important being the planar cell polarity (PCP) and the hedgehog signalling pathway (Hh).

Cell polarization refers to the organised establishment of asymmetries within cells. Just as intracellular functions are compartmentalised into organelles, many cellular functions are made more effective by partitioning along an axis of polarisation. Cell polarisation often leads to specific molecular determinants being localised to specific cellular domains, and the coordination of polarity in tissues is essential for the development of specialised forms and functions in multicellular organisms [16].

The Hedgehog (Hh) signalling pathway is an evolutionarily conserved signal transmission pathway from the cell membrane to the nucleus [17]. It plays an important role in normal embryonic development in invertebrates and vertebrates [18].

Some proteins are grouped to function as a whole forming complex, such as BBsome, and have been associated with ciliogenesis and cell polarity, and the hedgehog signalling pathway. The least known is the CPLANE (ciliogenesis and planar polarity effector) complex [19,20,21]. Defects in the genes encoding components of this complex have been related to failures of the ciliogenesis process and in planar cell polarity, thus associating the CPLANE complex with these processes [22]. However, the exact role of this complex is not well known, and the objective of this review is to collect information about the CPLANE complex and its function.

## 2. CPLANE Complex Structure

Throughout the combination of proteomic techniques, genetic analysis and in vivo imaging studies of proteins that have been linked to planar cell polarity, a set of Inturned (Inturned Planar Cell Polarity Protein), Fuzzy (Fuzzy Planar Cell Polarity Protein) and Wdpcp (WD Repeat Containing Planar Cell Polarity Effector) proteins, previously related to the ciliogenesis process [21,23], have been identified as a complex called CPLANE (ciliogenesis and planar cell polarity effector) [24]. These three elements are very closely linked to each other. In addition, it has been found that these three proteins interact with others that promote the assembly of the cilium. A clear interaction has been observed with the Jbts17 (Joubert Syndrome 17) protein (also call C5orf42) [24], previously related to Joubert syndrome and Orofacial–digital syndrome [25,26]. The interaction between the atypical Rab-type guanosine triphosphatase (GTPase) Rsg1 and Fuz was already known [20]. However, a recent study also found a link between Intu and Wdpcp with Rsg1 [24], suggesting that Intu favours the recruitment of Rsg1 in the late steps of ciliogenesis initiation [19,27].

The structure of the complex and the different interactions between the elements of the complex were practically unknown until now. A recent study carried out on mice and human cell lines has revealed that the complex adopts a crescent-like architecture, such that Wdpcp and Rsg1 are located at the ends of the crescent and bind to the central Intu-Fuz heterodimer on opposite sides. This creates a linear-type subunit interaction scheme that can be represented as Wdpcp-Intu-Fuz-Rsg1 [28].

Regarding the structure of the different proteins that conform the complex, Wdpcp (85.084 kDa) folds into a seven-bladed β-propeller belonging to the WD40 family, followed by an array of α helices [28]. Both Intu and Fuz have three longin-like domains (LD). LD1 of Intu (105.648 kDa) and Fuz (45.679 kDa) adopts a canonical longin fold, LD2 adopts a longin-like fold and LD3 adopts a lamp longin-like fold [29]. The core structure of Rsg1 (28.534 kDa) follows the typical small GTPase fold characterised by a central six-stranded concave β sheet surrounded by five α helices [28]. Wdpcp mainly contacts Intu with a large interface area of 2609 Å^2^. The main interaction platform is provided by Intu LD2, forming extensive contacts with the α-helical domain of Wdpcp. Intu contacts Fuz with a large buried surface area of 1898 Å^2^. Intu-Fuz heterodimer formation is mediated by LD1 and LD3 [28].

Nevertheless, it has been shown that Rsg1 interacts only with Fuz [28], and there is no interaction with the other elements of the complex as suspected [19,24]. The Fuz-Rsg1 interface spans an area of 1099 Å^2^ and consists of Fuz LD1 and LD2 engaging the central β sheet of Rsg1 [28].

## 3. CPLANE and Ciliogenesis

The relationship between the different elements that conform the CPLANE complex and the process of ciliogenesis has been extensively demonstrated.

First, disruption of the *FUZ* gene has been shown to disrupt ciliogenesis, resulting in shorter cilia, in *Xenopus* and mice [20,21,30]. However, in mice, cilia formation in different cell types such as Meckel’s cartilage cells, mesenchymal cells of the notochord and limb buds, and other cell types was not completely disrupted in the absence of *FUZ*, suggesting that other PCP effector genes, such as *INTU* [31] or *WDPCP*, may be involved in this process and compensate for the absence of *FUZ* [32].

In contrast, it was observed that the reduction of the amount of Intu protein by morpholino in *Xenopus* leads to the inefficient formation of cilia in the spinal cord and epidermis [21]. Furthermore, in *INTU* null mutant mouse embryos, primary cilia are observed in most ganglion cells. However, many of these cilia are severely atrophied, suggesting that *INTU* is not required for cilia formation per se, but is required for the formation of morphologically normal ganglion cilia [31]. Additionally, in fibroblast cell cultures, it was observed that there is no cilia formation in *INTU* null mutants [31]. All these data suggest that a clear link exists between *INTU* and the process of ciliogenesis.

Less data are available on the relationship between *WDPCP* and ciliogenesis; however, it has been observed that *WDPCP* is required for the recruitment to the ciliary transition zone of Mks1, Sept2 and Nphp1, three proteins required for ciliogenesis [33], and therefore there appears to be a relationship between *WDPCP* and ciliogenesis.

Finally, in a recent study carried out on mice, the effect of *RSG1* on cilia formation has been observed. Although most cells in the mesenchyme of the limb and adjacent to the neural tube are ciliated in the wildtype, fewer than 30% of mesenchymal cells in *RSG1* mutant mice embryos had cilia [19]. However, that 30% have normal cilia lengths, suggesting that the function of *RSG1* is somehow involved in increasing the efficiency of primary cilia initiation. In this study, the recruitment of Rsg1 to the mother centriole was confirmed to depend on its GTPase activity. Intu, which directly interacts with Rsg1 [24], was detected in *RSG1* mutants. Similarly, Ttbk2, a ciliogenesis-initiating protein, was detected in *INTU* mutants and *RSG1* mutants. In this way, it is proposed that Ttbk2 favours the recruitment of Intu, and this of Rsg1, acting Rsg1 in the last steps of the beginning of ciliogenesis [19]. However, as discussed above, a recent study has shown that Rsg1 only interacts with the CPLANE complex via Fuz [28], so the recruitment mechanism of Rsg1 in ciliogenesis remains unknown.

## 4. CPLANE and Ciliopathies

Ciliopathies comprise a heterogeneous group of genetic disorders caused by an alteration, which may be functional or structural, of the cilia [1,34].

As previously said, primary cilia are present in many tissues and cell types, which explains the wide range of clinical features associated with these disorders. Although these phenotypes can be related to organ-specific diseases, most ciliopathies are pleiotropic syndromes characterised by showing overlapping phenotypes [1,35]. Frequent cilia-related manifestations are (poly)cystic kidney disease, retinal degeneration, situs inversus, cardiac defects, polydactyly, other skeletal abnormalities, and defects of the central and peripheral nervous system, occurring either isolated or as part of syndromes.

There is a close relationship between the CPLANE complex and proper cilia formation and function. Thus, it is to be expected that mutations in the different components of the complex will lead to a malformation of the cilium and defects in its function, giving rise to ciliopathies. Here, we will try to gather the existing information on the relationship between the genes that make up the complex with the appearance of some ciliopathy.

First, dominant mutations in *FUZ* have been reported to cause isolated neural tube defects [36]. Another study showed that a frameshift deletion mutation in *FUZ* is associated with the development of short rib and polydactyly syndromes (SRPS) [37] (Table 1). The SRPS group includes six distinct autosomal recessive conditions, including four lethal conditions (Saldino-Noonan syndrome or SRPtype I (OMIM 263530), Majewski syn-drome or SRP type II (OMIM 263520), Verma–Naumoff syndrome or SRPtype III (OMIM 263510), and Beemer–Langer syndrome or SRPtype IV (OMIM 26986), and two that are compatible with life: EVC (OMIM 225500) and Jeune syndrome (ATD, OMIM 611263, OMIM 613091, OMIM 613819, and OMIM 614376). They are characterised by micromelia, short ribs, hypoplastic thorax, polydactyly (pre- and postaxial), and multiple anomalies of major organs [38].

In a recent study, null mutations in the PCP effector gene, *FUZZY*, were observed to cause profound early renal hypoplasia in mice [39].

For *INTU*, null mutant mice are homozygous lethal at mid-gestation and have been associated with the development of severe polydactyly [31]. In humans, a link has been found between mutations in *INTU* and the occurrence of SRPS [24]. Oral–facial-digital (OFD) syndromes are rare genetic disorders characterised by the association of abnormalities of the face (hypertelorism and low-set ears), oral cavity (lingual hamartoma, abnormal frenulum and lobulated tongue) and extremities (brachydactyly and polydactyly). OFD syndromes also comprise a broad range of additional features that initially led to the clinical delineation of 17 OFD subtypes [40]. Mutations in *INTU* are associated with the ciliopathy phenotype in the OFD type VI syndrome spectrum (OMIM 277170) [41]. Additionally, in another study, *INTU* was proposed as a possible cause of OFD type II syndrome (OMIM 252100) [40] and finally, pathogenic variants of *INTU* have been reported in two patients with OFDS XVII (OMIM 617926) [42] (Table 1).

**Table 1:** Ciliopathies related to CPLANE complex genes.

Gene	Disease	Reference
*INTU*	Short-Rib Polydactyly Syndrome (SRPS)	[24]
	Nephronophthisis	[24]
	Oro-facial-Digital Syndrome Type 2? (OFD2)	[40]
	Oro-facial-Digital Syndrome Type 17 (OFD17)	[42]
	Oro-facial-Digital Syndrome Type 6 (OFD6)	[41]
*FUZ*	Short-Rib Polydactyly Syndrome (SRPS)	[37]
*WDPCP*	Bardet-Biedl Syndrome (BBS)	[23]
	Meckel-Gruber syndrome (MKS)	[33]
*JBTS17*	Oro-facial-Digital Syndrome Type 6 (OFD6)	[25]
	Joubert Syndrome (JS)	[24]
	Meckel-Gruber syndrome (MKS)	[43]

All associations between genes and different pathologies have been observed in humans.

On the other hand, variants in WDPCP have been mainly related to OFDS [40]. It was found that WDPCP loss-of-function mutations may be sufficient to cause Bardet–Bield Syndrome (BBS, OMIM 209900) [23,44]. BBS is a pleiotropic ciliopathy characterised by six main clinical features: retinal dystrophy, truncal obesity, postaxial polydactyly, urogenital anomalies, renal abnormalities and cognitive impairment [45,46]. A wide range of secondary manifestations has been also reported. Mutations in WDPCP cause phenotypes similar to those found in individuals affected by Meckel-Gruber syndrome. (MKS, OMIM 249,000) [33] (Table 1). MKS is a lethal developmental syndrome characterised by posterior fossa abnormalities, bilateral enlarged cystic kidneys, and hepatic developmental defects that include ductal plate malformation associated with hepatic fibrosis and cysts. A common additional feature is postaxial polydactyly, usually affecting both hands and feet; occasional features are described [47].

Within the CPLANE complex, the gene that, until now, had a greater relationship with the appearance of ciliopathies is *JBTS17* (Table 1). To date, a large number of different pathogenic *JBTS17* mutations have been reported (mostly in patients with Joubert syndrome (JS) but a small part in OFDVI patients) [25,48,49,50]. JS is diagnosed by a pathognomonic malformation of the midbrain–hindbrain junction which results in the brain imaging finding “molar tooth sign” (MTS). Variable involvement of other organs (such as the eye, kidney, liver, and skeleton) is present in two-thirds of individuals with JS and can manifest at different ages and with variable severity [51]. In two more recent studies, new variants in *JBTS17* associated with JS have been identified [52,53]. Additionally, *JBTS17* has been associated with MKS [43].

The Small GTPase Rsg1 is the only component of the CPLANE complex for which no variant associated with the appearance of any ciliopathy has yet been described.

## 5. How Does the CPLANE Complex Relate to the Signalling Pathways Required for Ciliogenesis?

As mentioned above, the development of ciliopathies is due to failures during cilium formation, and we have seen the relationship between the CPLANE complex and ciliogenesis. More specifically, ciliogenesis depends on processes and signalling pathways such as intraflagellar transport, planar cell polarity or the Hedgehog signalling pathway, showing a link between these processes and the CPLANE complex.

### 5.1. Intraflagellar Transport

Ciliogenesis and cilia-mediated signalling both require the function of a highly conserved system of intraflagellar transport (IFT). The function of this system is to transport ciliary cargoes by using specific kinesin and dynein motors to move bidirectionally along the microtubule doublets of the ciliary axoneme [13,54]. Several genetic and biochemical studies have shown that the IFT is subdivided into two complexes: IFT-B, which governs the anterograde traffic from the cell body to the distal end of the axoneme, and IFT-A, which governs the retrograde return [13,55]. Even partial loss of IFT-B or IFT-A generally leads to impaired function of the entire subcomplex and loss of anterograde or retrograde functionality, respectively [56,57]. IFT has been shown to control cilia biogenesis and function in most ciliated eukaryotes, including vertebrates [58].

**Figure 1:**
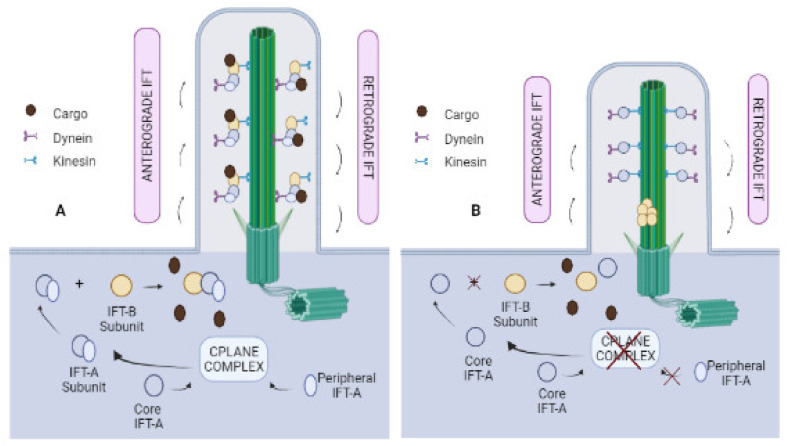
Relationship between the CPLANE complex and IFT. (**A**) Normal operation of the IFT when the CPLANE complex is undamaged; (**B**) IFT is truncated when the CPLANE complex is inoperative, generating shorter cilia.

The CPLANE complex and IFT have been related, mainly with IFT-A. In the absence of the CPLANE complex, peripheral IFT-A proteins do not localise to basal bodies and do not assemble into the core of the IFT-A subcomplex [24] (Figure 1). Moreover, it has been observed that CPLANE does not move along axonemes, thus showing that it is not a component of the IFT particle itself. This fact seems logical, since very strong relationships have been observed between the proteins that form the complex, but a weak relationship has been observed between CPLANE proteins and IFT proteins [24].

Focusing on the different components of the CPLANE complex, the first indication that CPLANE proteins play an important role in IFT came from the observation that *FUZ* disruption specifically affected protein localisation in the distal, but not the proximal, axoneme in *Xenopus* multiciliated cells [20]. In another study, an effect of *FUZ* on intraflagellar transport was observed. Fuz protein is required for proper localisation of retrograde IFT proteins within the cytoplasm, thus allowing maintenance of the cilium [59].

As discussed above, Fuz interacts directly with Rsg1 [28], so an effect of Rsg1 on intraflagellar transport seems likely. Knockdown (KD) of *RSG1* function has been shown to lead to similar, but not identical, defects in axonemal IFT dynamics compared to the loss of *FUZ*. KD of *RSG1* has also been shown to lead to defects in cytoplasmic IFT organisation which are similar to those observed following *FUZ* disruption, and to disorganisation of apically located basal bodies. However, the latter phenotype is not observed under *FUZ* KD conditions [27].

For other components of the CPLANE complex, another study confirmed the effect of *JBTS17* on intraflagellar transport. *JBTS17* knockdown specifically disrupted the recruitment of IFT-A subunits from the peripheral zone to the basal body. The levels of Ift139, Ift121 and Ift43 in the basal bodies were dramatically reduced after *JBTS17* knockdown [24].

Regarding the effect of the CPLANE complex on the IFT-B subunit, it has been observed that disturbance of either *JBTS17* or *WDPCP* disrupted IFT-B movement [24], as it happens with *FUZ* [59].

These data argue that CPLANE facilitates IFT-A recruitment and assembly at basal bodies [24]. However, it is surprising that the role of *INTU* in intraflagellar transport is not known, just as *WDPCP*, which information in this regard is scarce. In this way, considering the clear effect of the rest of the components of the CPLANE complex on intraflagellar transport, it would be interesting to carry out future research on the effect of *INTU* and *WDPCP* on IFT.

### 5.2. Planar Cell Polarity

The planar cell polarity (PCP) cascade is a conserved signalling pathway that governs the polarisation of cells within the plane of a cell sheet [21].

PCP genes have been extensively studied in *Drosophila melanogaster*. In this regard, *Drosophila* PCP genes are grouped into core PCP genes and tissue-specific PCP effector genes. The genes that are encompassed in central PCP are *FRIZZLED* (fz), *DISHEVELLED* (dsh), *PRICKLE* (pk), *DIEGO* (dgo), *STRABISMUS* (stbm, or Van Gogh (Vang)) and *FLAMINGO* (fmi, or starry night (stan)). PCP effector genes include *INTURNED* (in), *FUZZY* (fy), *FRITZ* (*WDPCP*, frtz), and *NEMO* (nmo) [60,61,62]. In vertebrates, PCP genes (*INTURNED*, *FUZZY* and *WDPCP*) are involved in a variety of functions, including neural tube closure, inner ear sensory cell hair bundle orientation and hair follicle orientation [63]. It has been shown that disruption of core PCP genes often results in severe defects in these processes [63,64,65], whereas mutations in PCP effector genes generally result in more subtle defects [21,66]. It is therefore proposed that, unlike PCP core genes, PCP effector genes may act in a more localised manner [67]. In addition, some vertebrate animals, like mammals, have acquired more sophisticated developmental processes, as more complex organogenesis and have even more PCP genes than *Drosophila* [63], supporting the theory that different PCP components are involved in different developmental processes in a tissue- or organ-specific manner.

The effect of genes belonging to the CPLANE complex on PCP has been studied. Disruption of *FUZ*, a PCP effector gene, resulted in delayed and arrested hair follicle development in mice, a phenotype not previously associated with PCP genes [67].

In another study carried out on *Xenopus laevis*, *INTURNED* and *FUZZY* were found to affect PCP signalling and cell intercalation [21], a fact that was expected because of their influence on planar cell polarity in the fly [22,66]. In particular, the directed elongation of microtubules is a critical aspect of both PCP in *D. melanogaster* and ciliogenesis [68]; because of this, it has been suggested that these proteins may influence the actin cytoskeleton during PCP in organising microtubules [21].

Finally, *Wdpcp*has been shown to modulate the actin cytoskeleton in mice by mediating the interaction of Sept2, a protein involved in the formation of cytoskeletal filaments, with actin. The formation of a Wdpcp-Sept2 complex may be required for Sept2 binding to and stabilisation of actin filaments. These results suggest that *WDPCP* may regulate planar cell polarity by modulating both the microfilament and microtubule cytoskeleton [33].

### 5.3. Hedgehog Signalling Pathway

The Hedgehog (Hh) signalling pathway is known to play an important role in embryonic development, organogenesis and tissue homeostasis [69]. In the adult stage, its activity is downregulated in most organs but can be reactivated in physiological and pathological processes such as tissue regeneration and cancer [70].

Numerous studies have long demonstrated the necessity of cilia for proper reception of the Hedgehog signal [71,72,73,74]. Activation of the Hh signalling pathway depends on the primary cilium, which is necessary for the translocation of Hh pathway components and the subsequent activation of Hh target genes, such as *GLI1* and *PTCH1* [75,76]. The importance of proper functioning of primary cilia for the Hh signalling pathway is well established for processes such as morphogenesis [3,77] or Hh-dependent tumour formation [78].

The effect of the CPLANE complex genes on the Hh signalling pathway has been extensively studied. It has been observed that up-regulation of *INTU* is necessary, but not sufficient, for ciliogenesis and oncogenic Hh signalling during basal cell carcinoma (BCC) formation [78]. *INTU* homozygous mutant mouse embryos show multiple defects, probably due to defective Hh signalling and *GLI3* processing [31]. In another study on mouse skin, suppression of Hh pathway activation was observed in *INTU* null mutant cells [32].

*INTURNED* and *FUZZY* function is required in the response to Hedgehog signals during early development in mammals [21]. In contrast, cilia are not required for Hedgehog signalling in *D. melanogaster* [79].

*FUZ* mutants in mice, generated by a gene-trap approach, exhibit multiple defects, including spinal cord and limb defects. In addition, these mutants have impaired Hh signalling and proteolytic processing of *GLI3*, which is consistent with the critical role of the Hh signalling pathway in the spinal cord and limb development. In this study, fewer cilia were observed in *FUZ* mutant mice, consistent with the key role of cilia in mediating Hh signalling in mammals [30]. Another study demonstrated the importance of *FUZ* in the formation of primary cilia in epidermal keratinocytes and dermal fibroblasts, both of which are required for Hh signalling during hair follicle morphogenesis [67].

Generation of a knockout mouse for *WDPCP* established that the CPLANE Wdpcp protein is essential for the development of the mouse appendicular skeleton, both in the limb bud and at later stages of skeletal development, including growth plate organisation. Significant alteration in hedgehog signalling activation was shown to be associated with defective proliferation and differentiation of skeletal tissues [80]. In *WDPCP* mutant MEFs was observed that Wdpcp deficiency disrupted Shh signalling, but in contrast, loss of Wdpcp function partially rescued the severe defect phenotypes of the *PTCH1* or *SMO* knockout embryos, which seems to indicate that Wdpcp may constrain Shh signalling downstream of Smo/Ptch1 [33].

The effect of *RSG1* on the Hedgehog pathway is not entirely clear. A recent study showed that the phenotypes of *RSG1* mutant mice could be caused by disruption of cilia-dependent Hh signalling. *GLI1*, a target of SHH signalling, was observed to be expressed at lower levels in the mutants, suggesting that *RSG1* acts downstream of SHH and upstream of *GLI1* in the central Hh signalling pathway [19]. As discussed above, *RSG1* mutants produce fewer hair cells, but these are of normal length. However, unlike the gene expression in WT MEFs, *GLI1* and *PTCH1* mRNA were not up-regulated in response to SAG in *RSG1* mutants. This suggests that although in the small number of mutant hair cells that form, Hh pathway activation is normal, this is not sufficient for normal Hh pathway activation in the cell population as a whole [19].

## 6. Conclusions

The primary cilium is a structure that can be found in most vertebrate cells and incorrect formation of the cilium can lead to ciliopathy. Several processes and signalling pathways are involved during ciliogenesis that are essential for proper function. Recent studies have confirmed a key role of the CPLANE complex in these processes. Mutations in the genes that conform the complex have been found to disturb the processes required during ciliogenesis, and the clinical features are related to ciliopathies. Given these data, further studies on the CPLANE complex are required, as it could play a key role in the development of certain ciliopathies.

## Data Availability

Not applicable.
